# Quantitative analysis of iridocorneal angle and ciliary cleft structures in canine eyes using ultrasound biomicroscopy

**DOI:** 10.3389/fvets.2024.1476746

**Published:** 2024-12-03

**Authors:** Donghee Kim, Hyun Kwon, Jiyi Hwang, Ji Seung Jung, Kyung-Mee Park

**Affiliations:** Laboratory of Veterinary Surgery and Ophthalmology, College of Veterinary Medicine, Chungbuk National University, Cheongju, Republic of Korea

**Keywords:** glaucoma, iridocorneal angle, ciliary cleft, ultrasound biomicroscopy, canine

## Abstract

**Introduction:**

This study aimed to examine the relationship between the relative opening of the ICA (RO-ICA) and the structure of the ciliary cleft (CC) using Ultrasound Biomicroscopy (UBM).

**Materials and methods:**

Clinical data from 31 eyes of 17 dogs at the Veterinary Teaching Hospital of Chungbuk National University, Korea, were analyzed. RO-ICA was categorized as “Slightly Narrow”, “Narrow”, “Open”, and “Wide Open”, with eyes further grouped into “Narrow” (including Slightly Narrow and Narrow) and “Open” (including Open and Wide Open) for analysis. Statistical methods, including linear regression and average comparisons between groups, were employed to explore correlations between RO-ICA and parameters such as ICA, CC width (CCW), CC length (CCL), and CC area (CCA).

**Results:**

The distribution showed “Narrow” (3 eyes, 9.7%), “Slightly Narrow” (13 eyes, 41.9%), and “Open” (14 eyes, 45.2%) as the predominant categories. In the Open group, CCL and CCA were significantly larger compared to the Narrow group. A positive correlation was observed between RO-ICA and CCL, and CCA across all groups. Notably, in the Narrow group, RO-ICA demonstrated a particularly significant positive correlation with all assessed parameters, including ICA, CCW, CCL, and CCA. However, no significant correlation was observed between RO-ICA and the assessed parameters in the Open group. In conclusion, while a smaller RO-ICA generally correlates with a smaller CC, a larger RO-ICA does not guarantee a larger CC.

**Conclusion:**

Additional UBM examinations are recommended for comprehensive evaluations, particularly in cases where gonioscopy indicates an open iridocorneal angle.

## 1 Introduction

Glaucoma in dogs is a serious, progressive disease that frequently leads to blindness (1, 2). This condition is characterized by the gradual narrowing and collapse of the iridocorneal angle (ICA), impairing the normal flow of aqueous humor and subsequently increasing intraocular pressure (IOP), a key contributor to vision loss (1, 3). Thorough evaluation of the ICA is critical in managing glaucoma. This integral component of the anterior chamber of the eye is responsible for regulating ocular fluid dynamics and intraocular pressure. Its alterations significantly contribute to the elevation of IOP and the progression of the disease (3–6). Therefore, precise assessment of the ICA and its related structures is imperative for the early detection and effective treatment of glaucoma in dogs.

Clinically, the assessment of the ICA in veterinary practice has predominantly relied on tools like gonioscopy or contact retinal camera (RetCam Shuttle; Clarity Medical Systems, Pleasanton, CA) for visual examination of the ICA’s anterior face (7–9). Gonioscopy, when feasible, reveals details such as narrow or closed iridocorneal angles, occasionally accompanied by pectinate ligament dysplasia (PLD) (9–11). However, this method has limitations due to its focus exclusively on the anterior aspect of the ICA, particularly in its inability to accurately measure the angle and evaluate finer structures such as the ciliary cleft (CC) (12). In contrast to gonioscopy, Ultrasound Biomicroscopy (UBM) provides a non-invasive and more precise approach (12, 13). It facilitates detailed imaging of the CC, an internal structure of the ICA, going beyond the capabilities of gonioscopy (14, 15). By providing cross-sectional images of the ICA, UBM emerges as an essential tool for accurate detection and effective management of ocular diseases like glaucoma (16, 17).

The ICA constitutes the peripheral, circumferential part of the anterior chamber, formed where the cornea, sclera, and base of the iris meet (4, 18). The anterior face of the ICA is composed of pectinate ligaments, characterized by slender, branching beams of iris tissue that span across the ICA, creating the iconic pectinate ligament structure (19–21). Posterior to the ICA lies the CC, a peripheral circumferential space extending posteriorly to the pectinate ligaments into the posterior ciliary body. This space, almost virtual in nature, forms a triangular shape with an anterior base (22–24). It serves as a crucial pathway for aqueous humor flow, connecting the radial collector channels that lead to the intrascleral venous plexus in the conventional outflow pathway, and to the porosity of the loose connective tissue and its extracellular matrix in the ciliary body in the unconventional outflow pathway (25–28). Therefore, abnormalities or changes in these structures can lead to pathologies and issues in intraocular pressure regulation (3, 5, 29).

Due to the significance of the ICA in primary angle closure glaucoma (PACG), considerable research has been conducted on the pectinate ligament through gonioscopy. These studies suggest that the narrowing of the ICA and PLD may play a role in PACG (3, 11, 30). In one particular study, the total outflow capacity, as measured by tonography, appeared normal when PLD was observed via gonioscopy. This implies that the condition of the pectinate ligament may present minimal resistance to outflow, especially if flow holes are present to allow aqueous humor access to the CC (29). Such findings highlight a potential discrepancy between the anterior aspect of the ICA, primarily the pectinate ligament as seen through gonioscopy, and its internal structure, the CC (12, 29, 31). Furthermore, this casts doubt on the reliability of using gonioscopy alone as an indicator to estimate total outflow capacity (16, 21, 32). This situation emphasizes the necessity for more in-depth research to investigate the correlation between these structural variances.

In our study, we aim to explore the relationship between the relative opening of the ICA and the structure of the CC using UBM. Our objectives include providing a more precise and quantifiable analysis of these structures. This will enable us to assess the extent to which the CC can be accurately predicted through clinically convenient methods like gonioscopy. This approach is anticipated to deepen our understanding of the interplay between the anterior and internal structures of the ICA, especially in the context of PACG. To our knowledge, this study is the first to quantitatively address the correlation between the relative opening of the ICA and the structure of the CC.

## 2 Materials and methods

### 2.1 Clinical information

This retrospective study utilized clinical data from dogs treated at the Veterinary Teaching Hospital of Chungbuk National University, Chungju, Korea. The data compilation spanned from August 29, 2018, to September 20, 2022, involving 31 eyes from 17 different dogs. Ethical approval for this research was granted by the Institutional Animal Care and Use Committee (CBNUA-1700-22-02). The ophthalmological evaluations were performed by Dr. KM Park, a veterinary medicine faculty member, along with a team of eye care veterinarians. These assessments included various tests, such as slit-lamp biomicroscopy (MW50D, SHIGIYA, Hiroshima, Japan), the Schirmer Tear Test (Schirmer Tear Flow Strips, GuldenOphthalmics, PA), assessments of the menace response, pupillary light reflex, and dazzle reflex, and rebound tonometry (TonoVet plus^®^, icare, Vantaa, Finland). Additional diagnostic methods encompassed gonioscopy (Ocular Koeppe Diagnostic Lenses, Ocular Instruments.inc), indirect ophthalmoscopy (Pan Retinal^®^ 2.2, VOLK, OH), and UBM (VuPAD^®^, Sonomed Escalon, Lake Success, NY, United States). In this research, patients exhibiting other ocular diseases aside from incipient cataracts were excluded during the complete ophthalmic examination process.

### 2.2 UBM examination

In this study, UBM examinations were essential to the ophthalmic assessments. To standardize the condition of all subjects with consistent pupil dilation, the dogs underwent topical application of 0.5% tropicamide (Mydriacyl^®^, Alcon, Geneva, Switzerland). UBM imaging was performed only after confirming full mydriasis. Additionally, 0.5% proparacaine hydrochloride (Alcaine^®^; Alcon) was applied for topical anesthesia. During the UBM procedure, the dogs’ eyelids were gently held open, and the UBM transducer was positioned precisely perpendicular to the corneoscleral limbus in the dorsal quadrant of the eye.

**Figure 1:**
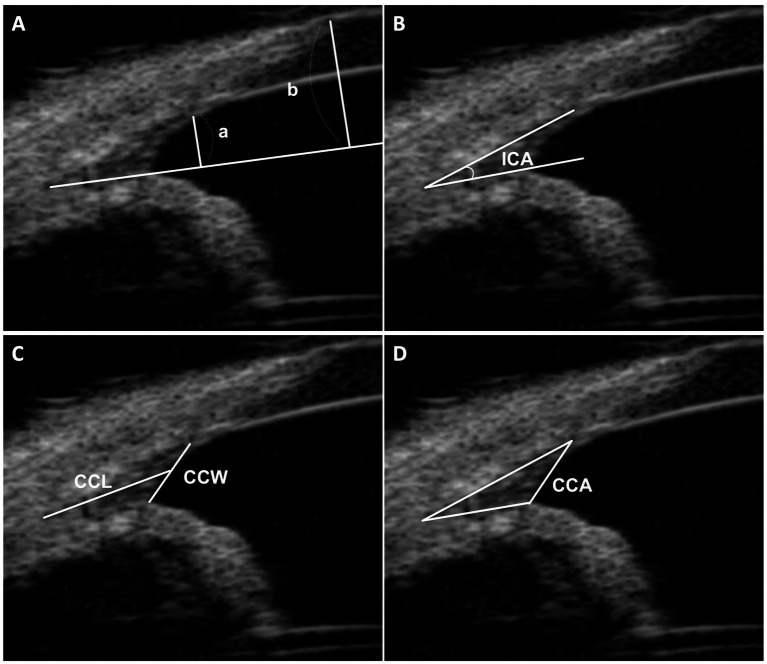
UBM measurement method. **(A)** This section illustrates the measurement of the relative opening of the iridocorneal angle (RO-ICA). It involves drawing a baseline from the lower surface of the ciliary cleft, extending the iris root line. Perpendicular lines are then drawn from the junction of the pectinate ligaments and sclera (length “a”) and from the anterior surface of the cornea (length “b”). The RO-ICA is calculated by dividing length “a” by length “b”. **(B)** Demonstrates the measurement of the Iridocorneal Angle (ICA), defined as the peripheral circumference of the anterior chamber at the convergence point of the sclera, cornea, and iris base. **(C)** Details the measurements of the ciliary cleft width (CCW) and length (CCL), with the CCW being the distance from the iris root to the corneoscleral, and the CCL measured from the angle recess to the midpoint of the CCW. **(D)** Describes the measurement of the Ciliary Cleft Area (CCA), which is the area enclosed by the ciliary cleft.

In our research, we adopted a method inspired by Ekesten et al., but specifically adapted for UBM imaging, to assess the relative opening of the iridocorneal angle (RO-ICA) (10, 11). This involved establishing a baseline along the lower edge of the CC, essentially extending the iris root line. Key measurements were taken perpendicular to this baseline: the distance at the point where the pectinate ligaments intersect the sclera (length “a”), and the distance from the anterior surface of the cornea (length “b”). The RO-ICA was then calculated by dividing length “a” by length “b”, thereby quantifying the relative opening in terms of proportional lengths, instead of focusing on absolute length measurements (Figure 1A).

The evaluation of the CC in each eye included four parameters based on established research (22–24): (a) the geometric Iridocorneal Angle (ICA), the angle formed between the plane of the iris root and the posterior corneoscleral limbus; (b) the width of the entrance to the CC (CCW); (c) the length of the CC (CCL); and (d) the area of the CC as manually measured (CCA). These assessments allowed for a detailed analysis of the CC’s anatomical structure and its potential implications for canine ocular health (Figures 1B–D).

### 2.3 Grading of the relative opening of the iridocorneal angle

In our study, the grading of the RO-ICA was conducted using the numerical grade scale established by Ekesten et al. (10). The grading system categorizes the RO-ICA into five distinct classes based on specific numerical intervals. These are as follows: “Closed” for an RO-ICA of X ≤ 0.15, “Narrow” for 0.15 < X ≤ 0.30, “Slightly narrow” for 0.30 < X ≤ 0.45, “Open” for 0.45 < X ≤ 0.55, and “Wide Open” for X ≥ 0.55. The X was calculated by dividing the distance at the intersection of the pectinate ligaments and the sclera (length “a”) by the distance from the anterior surface of the cornea (length “b”).

For further analysis in our study, the categories of “Narrow” and “Slightly Narrow” within the grading of the RO-ICA were combined to form the “Narrow Group”. Similarly, the “Open” and “Wide Open” categories were grouped together to constitute the “Open Group”.

### 2.4 Statistical analysis

In this study, we conducted our statistical analyses using SPSS software (version 17.0; SPSS Inc., Chicago). To assess differences in breed, sex, and weight among the groups, we utilized the Chi-squared (χ^2^) test. The Shapiro–Wilk test was initially applied to evaluate the normality of our data. Upon confirming data normality, we then employed the *t*-test to compare the mean values between groups. This method was specifically used for analyzing the differences between the narrow and open groups categorized under the RO-ICA classification. Furthermore, to investigate the relationship between RO-ICA measurements and various parameters assessed in the study, a single linear regression analysis was performed. The interpretation of the correlation was standardized as follows: R = 0.00–0.10, negligible correlation; R = 0.10–0.39, weak correlation; R = 0.40–0.69, moderate correlation; R = 0.70–0.89, strong correlation; R = 0.90–1.00, very strong correlation. We established levels of statistical significance at *p* < 0.05 represented as * in our study. For ease of interpretation in the figures, asterisks are used, whereas the actual *p*-values are explicitly detailed in the text of the study, ensuring comprehensive and clear communication of our statistical findings.

## 3 Results

### 3.1 The distribution of the relative opening of the iridocorneal angle

In the evaluation of the iridocorneal angles among the canine subjects, a variation in the RO-ICA grades was observed. The “Narrow” category comprised 3 eyes, accounting for 9.7% of the total. A significant portion, 41.9%, fell into the “Slightly Narrow” category, involving 13 eyes. The majority of the eyes, 14 in number, representing 45.2%, were classified as “Open”. The “Wide Open” category was the least represented, with only 1 eye, constituting 3.2% of the sample.

### 3.2 Canine characteristics

In the Narrow group, consisting of 16 eyes from 8 dogs, the breeds most commonly observed were Poodles and Malteses. The gender distribution favored males, with 5 males compared to 3 females. The mean age of the dogs in this group was 9.46 ± 4.21 years (range, 1.5–15 years), and the mean weight was recorded at 4.87 ± 1.60 kg (range, 2.85–9.80 kg). The IOP in this group was 16.69 ± 2.18 mmHg (range, 13.00–20.00 mmHg).

The Open group included 15 eyes from 10 dogs, with breeds such as Poodles and Shih Tzus being predominant. This group had a larger number of female dogs, with 6 females and 4 males. The mean age in this group was 7.90 ± 3.19 years (range, 4.0–15.0 years), and the mean weight was 5.60 ± 2.08 kg (range, 3.5–9.7 kg). The IOP in this group was 15.93 ± 2.25 mmHg (range, 12.00–20.00 mmHg).

Statistical analysis revealed no significant differences in terms of sex, weight, age or IOP between the Narrow and Open groups, indicating a similar distribution of these characteristics across both groups (Supplementary Tables 1, 2).

### 3.3 Comparative analysis of ICA, CCW, CCL, and CCA between narrow and open groups

**Figure 2:**
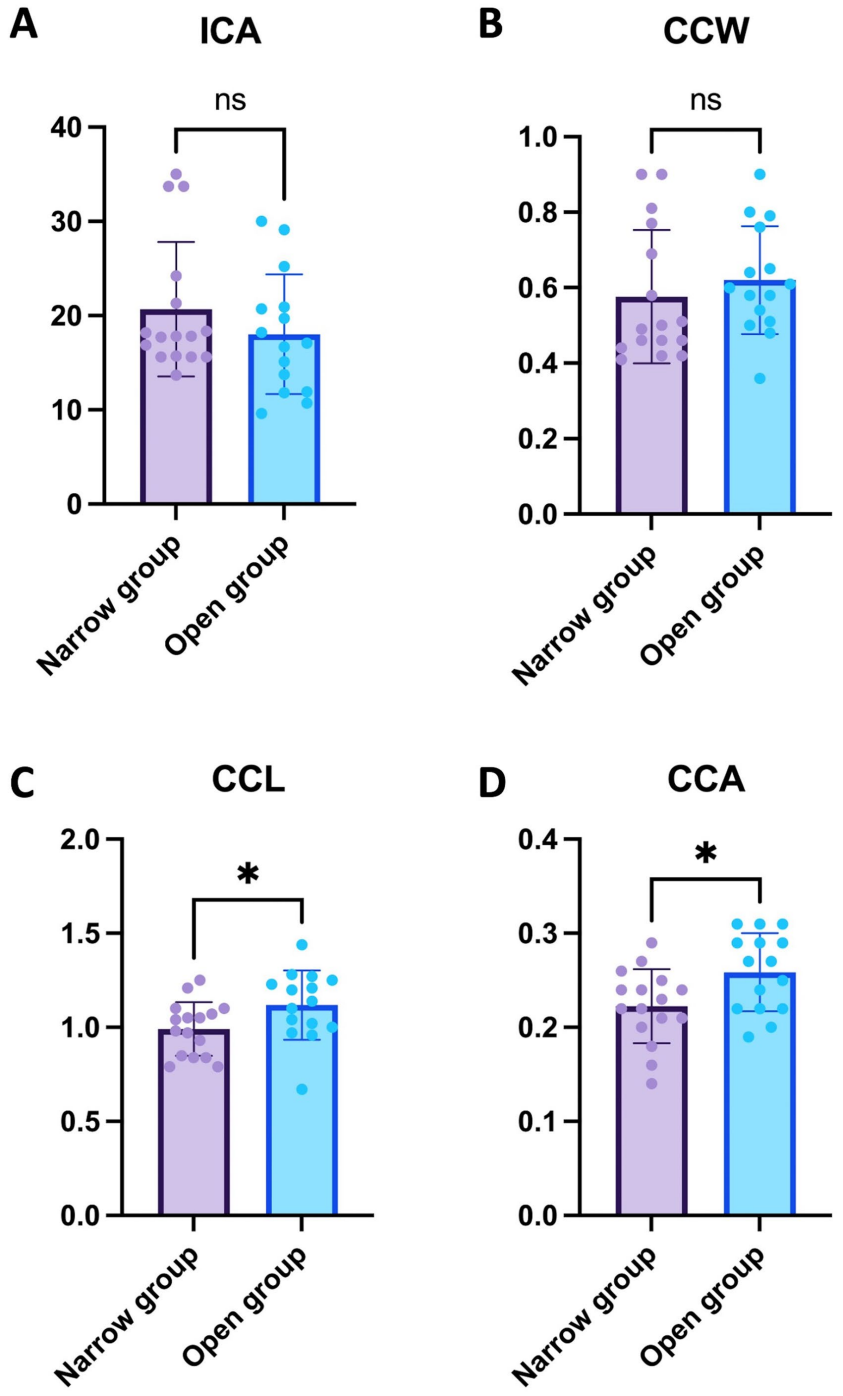
Comparative analysis of ICA, CCW, CCL, and CCA between narrow and open groups. **(A)** ICA Comparison in the Narrow group with 16 eyes showed a mean ICA of 20.68 ± 7.12°, in contrast to the Open group with 15 eyes at 18.03 ± 6.36°, with no statistically significant difference observed (*p* = 0.284). **(B)** CCW Comparison revealed mean values of 0.58 ± 0.18 mm in the narrow group of 16 eyes compared to 0.62 ± 0.14 mm in the open group of 15 eyes, without a significant difference (*p* = 0.464). **(C)** CCL Comparison indicated a significant difference, with the narrow group of 16 eyes showing a mean of 0.99 ± 0.14 mm and the open group of 15 eyes at 1.12 ± 0.18 mm (*p* < 0.05). **(D)** CCA Comparison also displayed a significant variation; the narrow group with 16 eyes had a mean CCA of 0.22 ± 0.04 mm^2^ compared to 0.26 ± 0.04 mm^2^ in the Open group of 15 eyes (*p* < 0.05). For clarity in interpreting our findings, levels of statistical significance in the study are denoted as follow: *p* < 0.05 (*).

In this study, the values of the ICA, CCW, CCL, and CCA were compared between the Narrow and Open groups. The mean ICA in the Narrow group was 20.68 ± 7.12°, while in the Open group, it was 18.03 ± 6.36°. This difference was not statistically significant, with a *p*-value of 0.284 (Figure 2A). Similarly, the CCW did not show a significant difference between groups, with mean values of 0.58 ± 0.18 mm in the Narrow group and 0.62 ± 0.14 mm in the Open group (*p* = 0.464) (Figure 2B). However, significant differences were observed in CCL and CCA measurements; the Narrow group exhibited a mean CCL of 0.99 ± 0.14 mm and CCA of 0.22 ± 0.04 mm^2^, whereas the Open group showed a mean CCL of 1.12 ± 0.18 mm and CCA of 0.26 ± 0.04 mm^2^ (*p* < 0.05, *p* < 0.05 respectively). These findings indicate that while the ICA and CCW do not significantly differ between the two groups, both CCL and CCA are notably larger in the Open group compared to the Narrow group (Figures 2C,D).

### 3.4 Regression analysis outcomes across combined narrow and open groups

**Figure 3:**
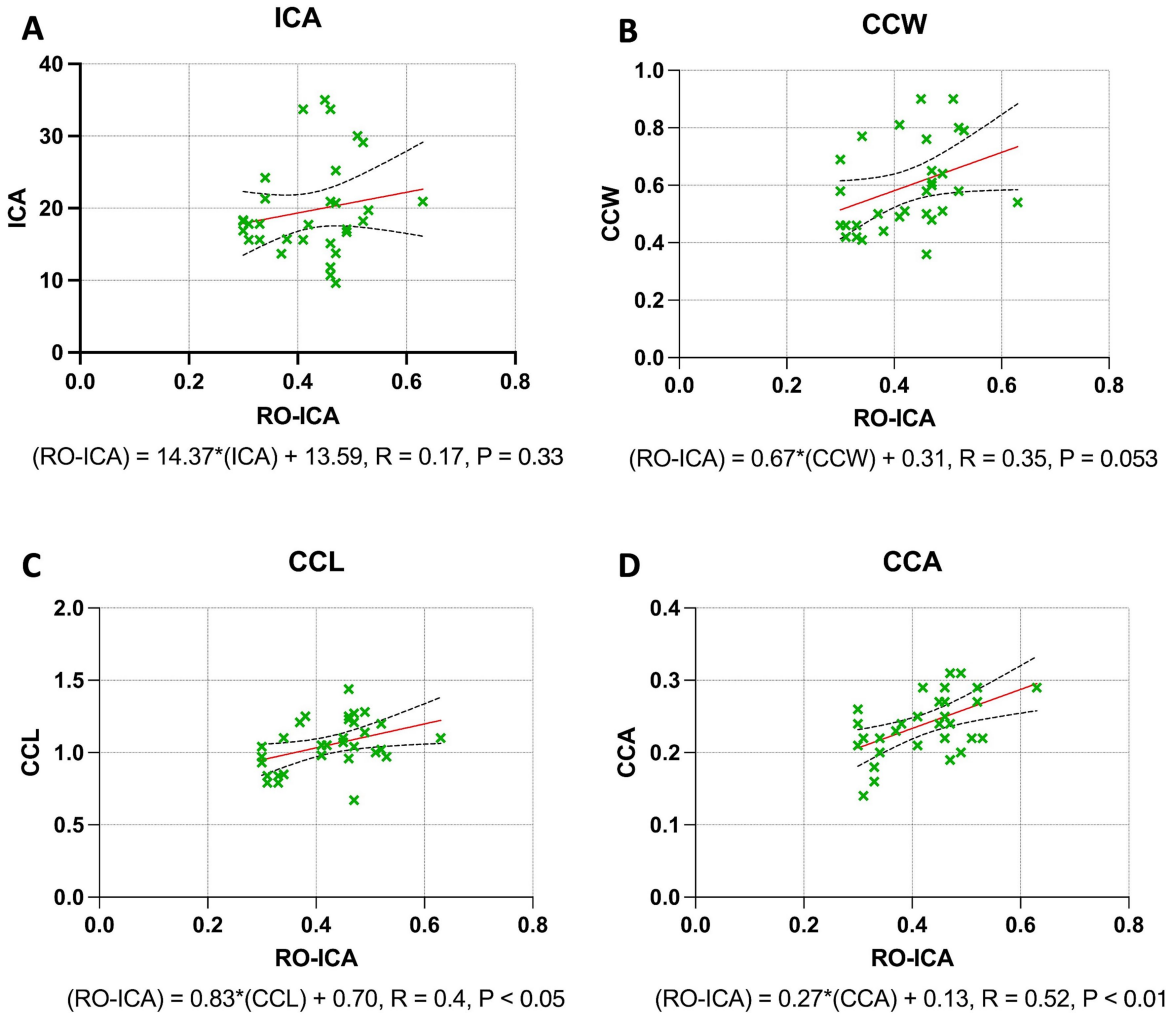
Regression analysis outcomes across combined narrow and open groups. **(A)** The linear regression model examining the relationship between RO-ICA and ICA in 31 eyes, described by the equation Y = 14.37X + 13.59, shows a weak linear correlation with an *R*^2^ value of 0.03316. Analysis of the slope and y-intercept, including standard errors and 95% confidence intervals, reveals no significant linear relationship (*p* = 0.3269). **(B)** The analysis of RO-ICA versus CCW in 31 eyes demonstrates a modest correlation using the equation Y = 0.6666X + 0.3144, resulting in an *R*^2^ of 0.1230. The confidence interval for the slope ranges from −0.009422 to 1.343, with a marginal slope significance (*p* = 0.0531), suggesting a weak linear relationship. **(C)** The linear regression model for RO-ICA versus CCL in 31 eyes, given by Y = 0.8282X + 0.7013, indicates a substantial correlation with an *R*^2^ of 0.1607. The slope is significantly non-zero (*p* < 0.05), confirming a significant linear relationship. Detailed confidence intervals for both slope and y-intercept are provided. **(D)** In assessing RO-ICA against CCA in 31 eyes, the model Y = 0.2698X + 0.1255 shows a moderate correlation with an *R*^2^ of 0.2684. A significant slope (*p* < 0.01) signifies a notable linear relationship. Standard errors and confidence intervals for both slope and y-intercept are included. Levels of statistical significance in the study are categorized as *p* < 0.05, *p* < 0.01, *p* < 0.001, and *p* < 0.0001 for clarity.

The regression analysis for the ICA yielded interesting insights. A positive correlation was observed between RO-ICA and ICA, but this association was not statistically significant. The regression model accounted for only 3.316% of the variance in ICA (*p* = 0.3269) (Figure 3A). Regarding the CCW, the analysis revealed a positive correlation with RO-ICA. However, the relationship was not statistically significant, with the model explaining 12.30% of the variance in CCW (*p* = 0.0531) (Figure 3B). For the CCL, the regression analysis showed a weak positive association with RO-ICA. This correlation was statistically significant, with the model accounting for 16.07% of the variance in CCL (*p* < 0.05) (Figure 3C). Lastly, in the case of the CCA, the regression analysis indicated a significant weak positive correlation with RO-ICA. The model explained a considerable 26.84% of the variance in CCA, and the association was statistically significant (*p* < 0.01) (Figure 3D).

### 3.5 Regression analysis outcomes in narrow groups

**Figure 4:**
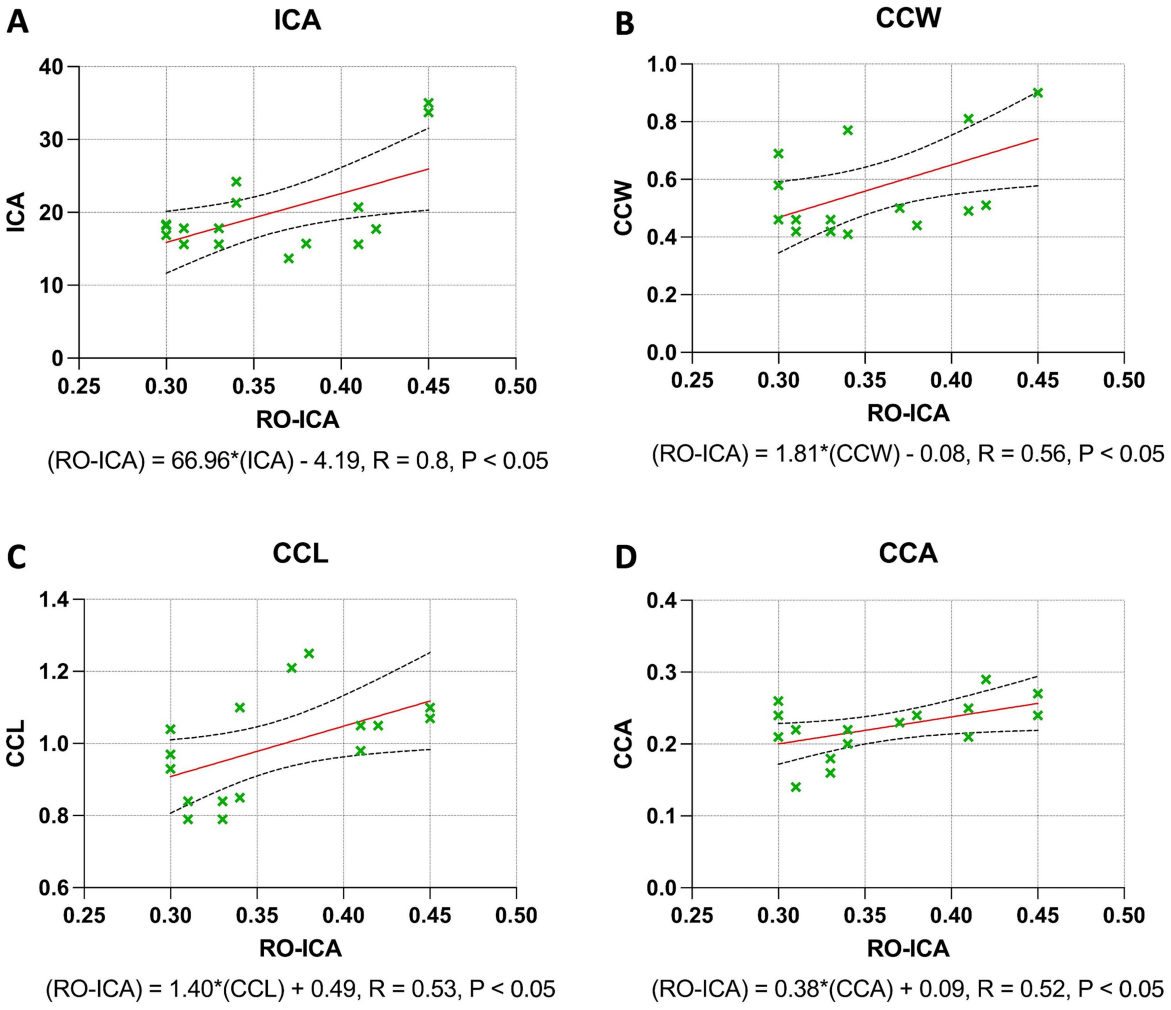
Regression analysis outcomes in narrow groups. **(A)** The regression model between RO-ICA and ICA for 16 eyes, represented by Y = 66.96X − 4.193, shows a moderate correlation with an *R*^2^ value of 0.3376. A significant slope (*p* < 0.05) supports a notable linear relationship. Standard errors and confidence intervals are provided for both slope and y-intercept. **(B)** In examining RO-ICA against CCW for 16 eyes, the equation Y = 1.816X − 0.07650 results in a correlation coefficient (*R*^2^) of 0.3080. The slope, significantly different from zero (*p* < 0.05), suggests a meaningful linear relationship. Confidence intervals for both slope and y-intercept are included. **(C)** The relationship between RO-ICA and CCL in 16 eyes is depicted by the linear model Y = 1.396X + 0.4894, demonstrating a fair correlation (*R*^2^ = 0.2794). The slope is significantly non-zero (*p* < 0.05), indicating a significant linear association. Detailed confidence intervals for the slope and y-intercept are provided. **(D)** The regression model for RO-ICA versus CCA in 16 eyes, given by Y = 0.3759X + 0.08741, exhibits a correlation of *R*^2^ = 0.2650. The significant slope (*p* < 0.05) implies a significant linear relationship. Standard errors and confidence intervals for both the slope and y-intercept are included in the figure. Levels of statistical significance in the study are categorized as *p* < 0.05, *p* < 0.01, *p* < 0.001, and *p* < 0.0001 for clarity.

The regression analysis for the ICA yielded significant insights. A moderate positive correlation was found between the RO-ICA and ICA, with the regression model explaining 33.76% of the variance in ICA. This association was statistically significant (*p* < 0.05), suggesting a strong linear dependence of ICA on RO-ICA (Figure 4A). Similarly, a weak positive correlation was observed with the CCW. The model accounted for 30.80% of the variance in CCW, and this relationship was statistically significant (*p* < 0.05), indicating a notable association between RO-ICA and CCW (Figure 4B). The CCL also showed a statistically significant weak positive association with RO-ICA, with the model explaining 27.94% of the variance in CCL (*p* < 0.05) (Figure 4C). Lastly, for the CCA, a significant weak positive correlation with RO-ICA was indicated. The model accounted for 26.50% of the variance in CCA, with this association being statistically significant (*p* < 0.05) (Figure 4D).

### 3.6 Regression analysis outcomes in open groups

**Figure 5:**
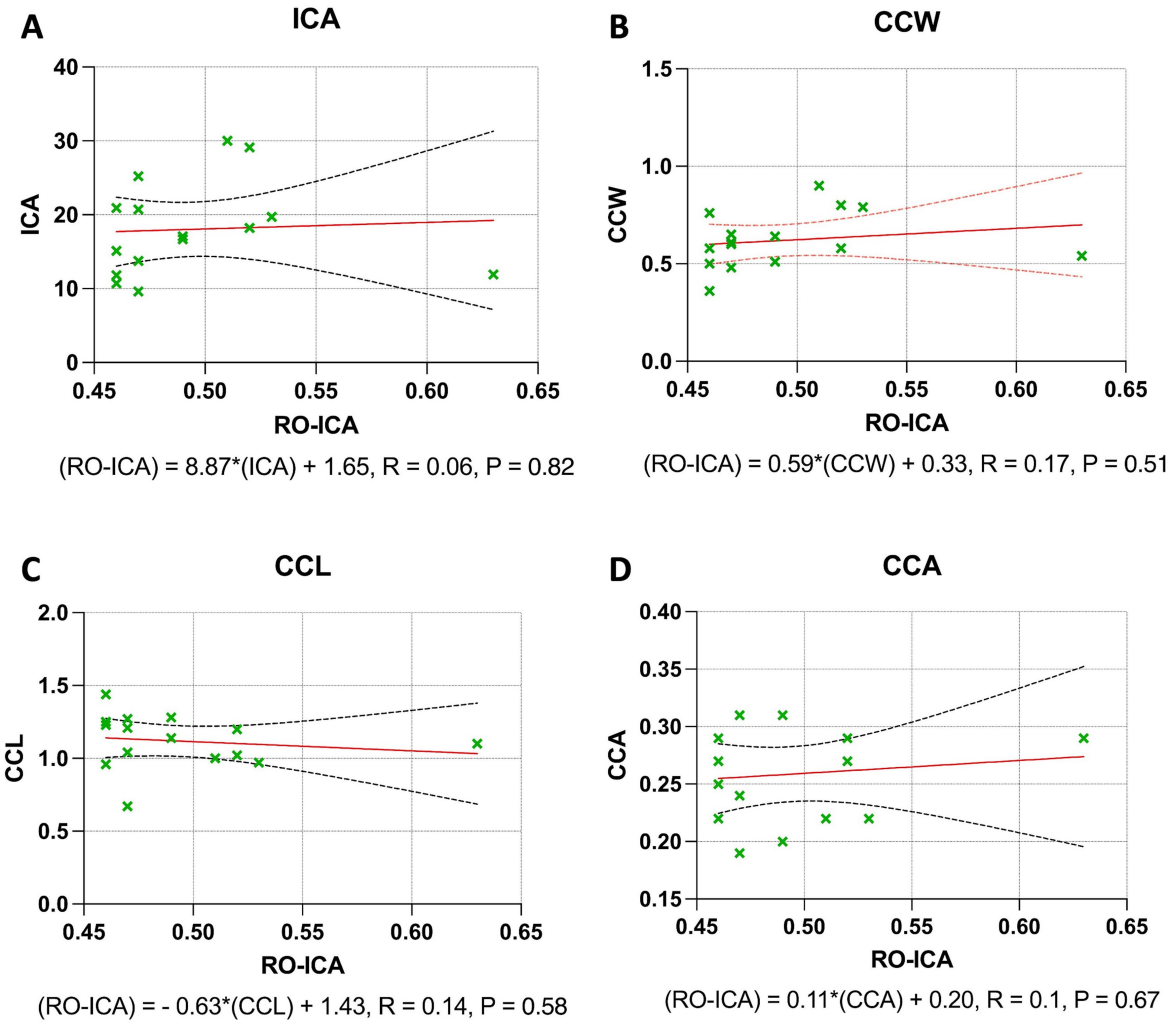
Regression analysis outcomes in open groups. **(A)** The linear regression model between RO-ICA and ICA for 15 eyes is represented by Y = 8.867X + 13.65, showing a very weak correlation with an *R*^2^ value of 0.003936. The large confidence intervals for the slope, ranging from −75.65 to 93.39, and y-intercept, from −28.26 to 55.56, demonstrate a high level of uncertainty. The slope’s insignificance (*p* = 0.8242) suggests no meaningful linear relationship. **(B)** For RO-ICA against CCW in 15 eyes, the regression equation Y = 0.5853X + 0.3308 results in a low correlation coefficient (*R*^2^ = 0.03416). The wide confidence interval of the slope, spanning from −1.279 to 2.450, and its non-significance (*p* = 0.5096) indicate a lack of significant linear relationship. **(C)** The relationship between RO-ICA and CCL in 15 eyes is depicted by Y = −0.6319X + 1.431, indicating a negligible correlation (*R*^2^ = 0.02378). The slope’s confidence interval, ranging from −3.058 to 1.794, along with its non-significance (*p* = 0.5832), implies no significant linear association. **(D)** The linear model for RO-ICA versus CCA in 15 eyes, given by Y = 0.1121X + 0.2033, exhibits minimal correlation (*R*^2^ = 0.01475). The slope, not significantly non-zero (*p* = 0.6663), with a confidence interval from −0.4370 to 0.6612, suggests a non-significant linear relationship. Levels of statistical significance in the study are categorized as *p* < 0.05, *p* < 0.01, *p* < 0.001, and *p* < 0.0001 for clarity.

In the Open group, the regression analysis for the ICA revealed that the association between RO-ICA and ICA was not statistically significant, with the model accounting for only 0.39% of the variance in ICA (*p* = 0.8242). This suggests a negligible linear dependence between RO-ICA and ICA (Figure 5A). Similarly, the CCW showed a non-significant relationship with RO-ICA, with the regression model explaining just 3.42% of the variance in CCW (*p* = 0.5096) (Figure 5B). For the CCL, the regression analysis indicated an equally minimal relationship, accounting for merely 2.38% of the variance in CCL (*p* = 0.5832), pointing to no strong linear correlation between RO-ICA and CCL (Figure 5C). Lastly, the analysis for the CCA also demonstrated a very weak linear relationship with RO-ICA, as the model accounted for only 1.48% of the variance in CCA (*p* = 0.6663) (Figure 5D).

## 4 Discussion

In this study, our goal was to investigate the correlation between the RO-ICA and the structure of the CC using UBM We aimed to provide a detailed, quantitative analysis of these structures, enabling a more accurate prediction of CC characteristics through methods like gonioscopy.

This study was conducted retrospectively, including all patients who visited during the specified period. During patient selection, those with ophthalmic diseases other than incipient cataracts were excluded, and no specific selection criteria were applied regarding breed. For cases in which both eyes met the measurement criteria, each eye was treated as an independent data point; for cases with only one eye meeting the criteria, only that eye was included in the analysis.

Previous studies have established a relationship between the progressive narrowing of the ICA and the development of glaucoma (3, 11). It has also been demonstrated that the risk of developing PACG is significantly higher in dogs with a narrowed or closed CC (29, 33). Our study sought to clarify the relationship between these two factors, which are considered critical. Overall, we found a positive correlation between the RO-ICA and the CC. However, we also demonstrated that, even in cases categorized as “Open” gonioscopically, there can still be a presence of a narrowed CC despite a relatively larger opening of the ICA. This finding challenges some of the traditional understandings of the relationship between ICA opening and CC in canine eyes (12).

The method used for measuring the RO-ICA in this study was developed through modifications of the techniques previously described by Ekesten et al. (10) and Bjerkås et al. (11). Unlike previous studies that relied on gonioscopy to observe and measure only the anterior face of the ICA, our research utilized UBM to examine sectioned images of the ICA. Additionally, we adopted the criteria for evaluating the measurement values directly from Ekesten et al.’s study (10). In contrast to previous research, where the percentages of Slightly Narrow, Narrow, Open, and Wide Open were 6.4, 25, 55.9, and 9.3% respectively, our study found these categories to be 9.7, 41.9, 45.2, and 3.2%. Notably, in our study, the proportion of eyes classified in the narrow group was higher than that in previous research. This difference could be attributed to the fact that the earlier study focused on Samoyeds, a single breed, while our study included a variety of breeds. Predominantly, breeds like Poodles and Shih Tzus, known to be prone to glaucoma due to structural reasons, were part of our research, which might explain the relatively higher proportion of the narrow group in our findings (34, 35).

Numerous risk factors for PACG have been identified in previous studies. Notably, female dogs are reported to have approximately twice the risk of developing PACG compared to males (36, 37). One theory suggests that gender differences in ICA morphology may contribute to this increased risk (16, 38, 39). Additionally, there has been an observation of progressive narrowing of the ICA with age, a finding that is significant as it implies that the width of the ICA may be a dynamic feature, changing as the dog ages, which could influence the risk of developing glaucoma (10, 21). There has also been a noted positive correlation between age and the severity of PLD (11, 21, 32). However, our study observed no significant differences in terms of sex, weight, age, and breed between the Narrow and Open groups.

Based on the results of this study, it was observed that the CCL and CCA were statistically significantly smaller in the Narrow group compared to the Open group. Additionally, when conducting regression analysis regardless of the group, both CCL and CCA demonstrated a positive correlation with the RO-ICA. This correlation was more pronounced when the ICA’s relative opening was smaller, particularly in the Narrow group. Here, all factors, including ICA, CCW, CCL, and CCA, showed a positive correlation with RO-ICA. In essence, this suggests that a smaller RO-ICA is likely to be associated with a smaller CC. These findings are in line with previous results that showed no significant difference between UBM-derived CC grades and subjective gonioscopic grades, further reinforcing the consistency and validity of these measures (12).

However, in the Open group, despite the CCL and CCA being significantly larger compared to the Narrow group, there was no observed correlation with the RO-ICA. This outcome contrasts with previous studies that found no differences between UBM-derived CC grades and subjective gonioscopic grades when the CC depicted on UBM images was graded as open (12). This result suggests that a large RO-ICA does not necessarily correlate with a large CC. This discovery holds substantial clinical importance, indicating that even if the ICA is assessed as open through gonioscopy, there could still be a predisposition to PACG. The findings also imply the potential necessity of evaluating the CC using UBM examinations.

Previous studies have recognized the CC as an essential anatomical structure in the formation of IOP. Research by Dubin et al. highlighted that a narrowed or closed CC in dogs significantly increases the risk of developing PACG by 20 times. Their findings also revealed that, despite the angle index assessed through gonioscopy providing a subjective estimate of outflow capacity through the iridocorneal angle, the pectinate ligament might not be the critical factor in restricting the flow of aqueous humor through the iridocorneal angle (33). Additionally, other studies have indicated that even in cases with PLD, if the CC remains open, the overall outflow capacity may still be normal (2, 29). Further research, particularly studies on goniotomy which involves widening the CC, has demonstrated an increase in the flow of aqueous humor, underscoring the CC’s importance in IOP regulation and its pivotal role in the pathogenesis of PACG (40).

One of the parameters studied in our research, the ICA, overall, did not exhibit a correlation with the RO-ICA. However, in the Narrow group, a positive correlation was observed, indicating that a smaller RO-ICA tends to correspond with a smaller ICA. Since gonioscopy cannot evaluate the internal structure of the ICA, making it an imperfect marker, there has been research attempting to understand their relationship (21). In previous studies, the ZibWest angle index, which comprehensively evaluates the grade of ICA and PLD, was used to assess the correlation with ICA. These studies revealed that when ICA measurements were conducted using SD-OCT, they showed significant correlations with gonioscopic ZibWest angle indices (41). Although PLD was not evaluated in our study, a similar outcome was observed in terms of the correlation between ICA grade and ICA, aligning with these prior findings.

This study has several limitations that should be acknowledged. A primary limitation is the small sample size, which may restrict the generalizability of our findings. While a pre-study power analysis was not conducted, we consider this study an initial observational effort to investigate the relationship between the relative opening of the iridocorneal angle (RO-ICA) and the ciliary cleft (CC) structure. A larger sample size would be necessary to confirm these findings and potentially identify broader correlations between RO-ICA and CC. Additionally, the method we used to calculate RO-ICA was originally developed for gonioscopy images. Consequently, there may be some differences when compared to cross-sectional imaging. This also applies to the standard values used for interpretation, which could vary. Despite these potential discrepancies, this study made a concerted effort to measure RO-ICA in a consistent and standardized manner. Furthermore, our study included cases of incipient cataract. While some research suggests that cataracts may influence the size of the ciliary cleft, we did not observe significant differences between normal eyes and those with incipient cataracts (24). As a result, we included these cases in our study results, considering them relevant to our overall findings.

**Figure 6:**
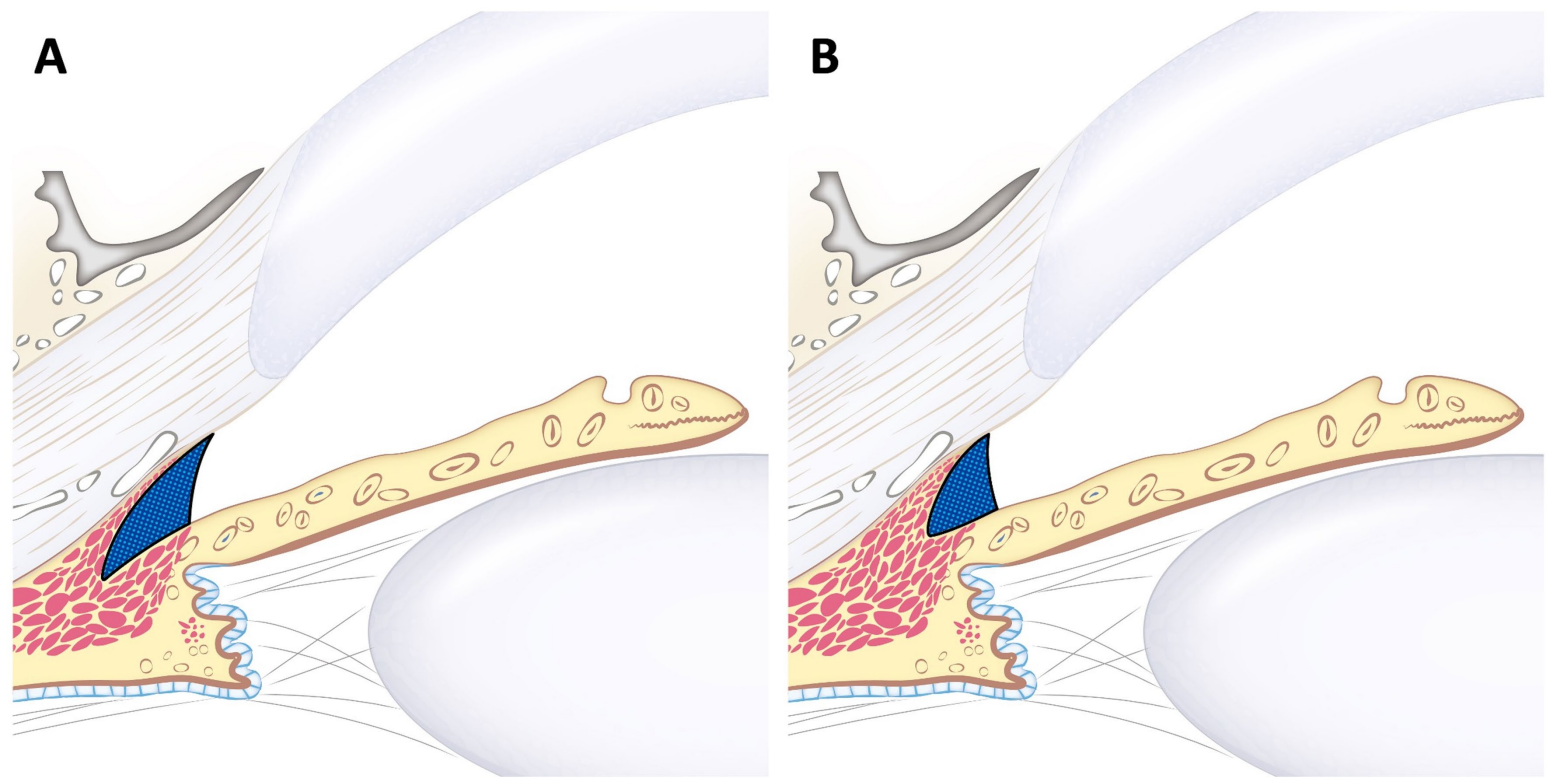
Schematic image of large RO-ICA with varying sizes of the ciliary cleft. This figure illustrates the different ciliary clefts in the same larger RO-ICA. Although **(A)** and **(B)** have the same relatively large RO-ICA, **(B)** shows that CCW, CCL, and CCA are all smaller compared to **(A)**. This indicates that even with the same relative opening, the ciliary cleft can vary in size.

In conclusion, a smaller RO-ICA can generally be associated with a smaller CC. However, a larger RO-ICA does not necessarily indicate a larger CC. Therefore, in clinical settings, when an open ICA is observed using gonioscopy, the possibility of a smaller CC should always be considered (Figure 6). Consequently, additional UBM examinations are recommended for a more comprehensive assessment.

## Data Availability

The original contributions presented in the study are included in the article/supplementary material, further inquiries can be directed to the corresponding author.
