# Supplementary material for: Quantitative analysis of iridocorneal angle and ciliary cleft structures in canine eyes using ultrasound biomicroscopy
Source: Front Vet Sci. 2024 Dec 3;11:1476746. doi: 10.3389/fvets.2024.1476746 (PMC11649674; doi:10.3389/fvets.2024.1476746)
Supplement: Supplementary file 1 [file Table_1.docx]

**Supplementary table 1. Summary of Narrow Group Population with Breed, Gender, Age, Laterality and IOP**

| **ID** | **Breed** | **Gender** | **Age** | **Laterality** | **IOP** |
| --- | --- | --- | --- | --- | --- |
| NG 1 | Chihuahua | IM | 11Y | OD | 19 |
| NG 1 | Chihuahua | IM | 11Y | OS | 19 |
| NG 2 | Poodle | SF | 15Y | OD | 16 |
| NG 2 | Poodle | SF | 15Y | OS | 17 |
| NG 3 | Mixed | CM | 1.5Y | OD | 19 |
| NG 3 | Mixed | CM | 1.5Y | OS | 17 |
| NG 4 | Poodle | SF | 6.28Y | OD | 13 |
| NG 4 | Poodle | SF | 6.28Y | OS | 14 |
| NG 5 | Maltese | CM | 11.4Y | OD | 13 |
| NG 5 | Maltese | CM | 11.4Y | OS | 20 |
| NG 6 | Chihuahua | IM | 10Y | OD | 17 |
| NG 6 | Chihuahua | IM | 10Y | OS | 19 |
| NG 7 | Poodle | SF | 13.5Y | OD | 17 |
| NG 7 | Poodle | SF | 13.5Y | OS | 16 |
| NG 8 | Maltese | CM | 7Y | OD | 16 |
| NG 8 | Maltese | CM | 7Y | OS | 15 |
