# Supplementary material for: Quantitative analysis of iridocorneal angle and ciliary cleft structures in canine eyes using ultrasound biomicroscopy
Source: Front Vet Sci. 2024 Dec 3;11:1476746. doi: 10.3389/fvets.2024.1476746 (PMC11649674; doi:10.3389/fvets.2024.1476746)
Supplement: Supplementary file 2 [file Table_2.docx]

**Supplementary table 2. Summary of Open Group Population with Breed, Gender, Age, Laterality and IOP**

| **ID** | **Breed** | **Gender** | **Age** | **Laterality** | **IOP** |
| --- | --- | --- | --- | --- | --- |
| OG 1 | Poodle | SF | 8Y | OD | 16 |
| OG 1 | Poodle | SF | 8Y | OS | 15 |
| OG 2 | Bichon frise | SF | 5.4Y | OD | 12 |
| OG 2 | Bichon frise | SF | 5.4Y | OS | 13 |
| OG 3 | Pomeranian | SF | 8Y | OD | 14 |
| OG 3 | Pomeranian | SF | 8Y | OS | 15 |
| OG 4 | Shih tzu | IF | 5.88Y | OD | 17 |
| OG 4 | Shih tzu | IF | 5.88Y | OS | 19 |
| OG 5 | Shih tzu | CM | 4Y | OD | 20 |
| OG 5 | Shih tzu | CM | 4Y | OS | 17 |
| OG 6 | Maltese | CM | 13Y | OD | 19 |
| OG 7 | Mixed | CM | 11Y | OS | 16 |
| OG 8 | Beagle | IF | 7Y | OD | 14 |
| OG 9 | Poodle | SF | 15Y | OD | 16 |
| OG 10 | Maltese | CM | 10Y | OS | 16 |
